# Effects of a 6-month aerobic exercise intervention on brain morphology in women with breast cancer receiving aromatase inhibitor therapy: a sub-study of the EPICC trial

**DOI:** 10.3389/fnhum.2024.1443916

**Published:** 2024-10-23

**Authors:** Cristina Molina-Hidalgo, Lu Wan, Daniel Velazquez-Diaz, Haiqing Huang, George Grove, Catherine M. Bender, Amanda L. Gentry, Susan M. Sereika, Chaeryon Kang, Mary E. Crisafio, Kirk I. Erickson

**Affiliations:** ^1^AdventHealth Research Institute, Neuroscience Institute, Orlando, FL, Unites States; ^2^Deparment of Psychology, University of Pittsburgh, Pittsburgh, PA, United States; ^3^School of Nursing, University of Pittsburgh, Pittsburgh, PA, United States; ^4^School of Nursing, School of Public Health, University of Pittsburgh, Pittsburgh, PA, United States; ^5^Department of Biostatistics, University of Pittsburgh, Pittsburgh, PA, United States

**Keywords:** breast cancer, aerobic exercise, brain morphology, cortical thickness, postmenopausal

## Abstract

**Objective:**

Physical exercise may increase brain volume and cortical thickness in late adulthood. However, few studies have examined the possibility for exercise to influence brain morphology in women treated for breast cancer. We conducted a nested sub-study within a randomized clinical trial to examine whether 6 months of moderate-intensity aerobic exercise in postmenopausal women with early-stage breast cancer influences brain morphology.

**Methods:**

We included twenty-eight postmenopausal women newly diagnosed with Stage 0-IIIa breast cancer (M age = 62.96 ± 5.40) who were randomized to either 45–60 min of supervised aerobic exercise 3 days/week (*n* = 16) or usual care (*n* = 12). Before beginning aromatase inhibitor aromatase inhibitor therapy, and the exercise intervention, and again at 6-month follow-up, volumetric and cortical thickness measures were derived from magnetic resonance imaging scans.

**Results:**

There were no significant intervention effects on brain volume and cortical thickness. However, greater average exercise intensity (%) during the intervention was associated with greater post-intervention cortical volume, mean cortical thickness, precentral gyrus thickness, and superior parietal thickness (all *p* < 0.05). Finally, total supervised exercise time was associated with higher precentral gyrus thickness after the intervention (*p* = 0.042, *R*^2^ = 0.263).

**Conclusion:**

The exercise intervention did not significantly affect brain volumes and cortical thickness compared to the control group. However, positive associations were found between exercise intensity and brain morphology changes after the 6-month intervention, indicating that exercise may reduce the vulnerability of the brain to the deleterious effects of breast cancer and its treatment.

## 1 Introduction

Breast cancer and breast cancer treatments are associated with altered brain structure and function, which are also linked to poorer cognitive performance, including memory and executive function (Ahles et al., 2012; Bender et al., 2015; Janelsins et al., 2014; Peukert et al., 2020). Previous studies have shown that structural brain alterations include global and regional grey matter volumetric reductions (Ahles et al., 2010; Lange et al., 2019), including changes in the temporal cortices (Kaiser et al., 2014). In this context, the hippocampus has gained much interest due to its connection with higher cortical brain structures and the limbic system (Schultz and Engelhardt, 2014). Numerous cross-sectional and longitudinal neuroimaging studies have observed that the hippocampus is more vulnerable to both structural and functional decline in the context of chemotherapy-exposed women with breast cancer (de Ruiter et al., 2011; Dubois et al., 2014; Inagaki et al., 2007; Peukert et al., 2020). For instance, several studies have reported that women with breast cancer exhibit smaller hippocampal volumes as compared to women without cancer (Peukert et al., 2020). These cancer-related changes in brain health have led to speculation about methods to prevent neurocognitive decline or to remediate brain health with intervention.

Physical exercise is one of the most promising non-pharmaceutical approaches for positively influencing brain health (Aghjayan et al., 2022; Aghjayan et al., 2021; Stillman and Erickson, 2018; Stillman et al., 2020). Recent reviews and meta-analyses demonstrate that aerobic exercise is an effective approach for improving self-reported cognitive function in women with breast cancer (Campbell et al., 2020; Koevoets et al., 2022; Ye Xin-Xin et al., 2022), and higher cardiorespiratory fitness levels are associated with greater hippocampal volumes in women treated for breast cancer (Chaddock-Heyman et al., 2015). Yet, despite these promising patterns of results, the effects of an exercise intervention on brain morphology in women with breast cancer remain infrequently studied and poorly understood.

In one 6-month randomized controlled trial (*N* = 118) investigating the effects of an exercise intervention on cognitive function and brain structure/function in physically inactive breast cancer patients, there were no exercise-related effects on the hippocampus, cortical thickness, or grey matter (GM) volume (Koevoets et al., 2023). In contrast, our main randomized clinical trial (RCT) demonstrated in 153 postmenopausal women with hormone receptor-positive early-stage breast cancer within two years post-primary breast cancer therapy that exercise improved measures of processing speed relative to the control group (Bender et al., 2024). Improvements were also found in learning, memory, and working memory function, and greater adherence to the intervention was associated with a greater improvement in cognitive performance. Yet, despite these cognitive improvements, there is a dearth of information on the consequences of an exercise intervention on brain morphology.

This nested sub-study was built logically from this prior research and was designed to explore, compared to usual care, the effects of a supervised 6-month moderate-intensity aerobic exercise intervention on brain volume and cortical thickness in 28 postmenopausal women with early-stage, hormone receptor-positive breast cancer. Additionally, we examined whether exercise-related increases in cardiorespiratory fitness were associated with increases in brain morphology. We predicted that exercise would increase the hippocampus and prefrontal cortex volumes compared to usual care and that those changes would be correlated with cardiorespiratory fitness improvements. Finally, in an exploratory analysis, we examined the association between changes in brain morphology and the total time of supervised exercise and average exercise intensity achieved during the supervised sessions.

## 2 Materials and methods

### 2.1 Participants and study design

The Exercise Program in Cancer and Cognition ([EPICC], Clinical Trials.gov NCT02793921) study was a randomized controlled trial in which 153 postmenopausal women with hormone receptor-positive, early-stage breast cancer were assigned to receive (i) a 6-month moderate-intensity aerobic exercise intervention (aerobic exercise group) or (ii) usual care (control group). Participants were recruited from medical and surgical oncology clinics of the UPMC Hillman Cancer Center Breast Cancer Program and the Breast Care Center of St. Clair Health (2015–2019). Ethics approval was obtained from the University of Pittsburgh Human Research Protection Office (PRO15120433), St. Clair Hospital IRB (PRO1712001), and the Carnegie Mellon University Institutional Review Board (study2016_00000197). Details concerning the inclusion and exclusion criteria have been previously described (Gentry et al., 2018). Briefly, eligible women were (i) postmenopausal, (ii) < 80 years of age, (iii) diagnosed with stage 0, 1, 2, or 3a breast cancer, (iv) within two years of completion of primary breast cancer therapy (surgery +/- chemotherapy) and (v) English-speaking with a minimum of 8 years of education. Women were excluded from participation if they received chemotherapy for a previous cancer, had clinical evidence of distant metastasis, were hospitalized for psychiatric illness within the previous two years, had any medical condition that would preclude exercise, had persistent breast cancer surgery complications, used an assisted walking device, or had a history of neurological illness, substance abuse, falls or balance problems. In addition, exclusion criteria included claustrophobia and the presence of irremovable metal implants incompatible with MRI. The participants for this analysis were among a subsample of women who were eligible for and consented to undergo MRI scanning (*n* = 28). All participants provided written informed consent.

### 2.2 Procedure

The randomization for the present sub-study was part of the parent EPICC study, but we did not stratify randomization for the parent study based on MR eligibility. After baseline comprehensive neuropsychological testing and a neuroimaging scan, women were randomly assigned to the aerobic exercise intervention or usual care control group using a minimization algorithm to balance study arms on age ( ≤ 60, > 60 to ≤ 70) and baseline executive function performance (composite mean z-scores, ≤ −0.5, > −0.5 to < +0.5, ≥ +0.5). Follow-up assessments (T2) occurred within two weeks of completion of the 6-month intervention and at a comparable time in the control group.

### 2.3 Intervention

Consistent with the American College of Sports Medicine (ACSM) recommendations for cancer survivors, the 6-month exercise intervention prescribed a minimum of 150 min of moderate-intensity aerobic exercise each week (Campbell et al., 2019; Patel et al., 2019). All supervised exercise sessions began and ended with a vital sign check and a 5 to 10-min warm-up and cool-down. Most women started with 10 to 15 min of moderate-intensity exercise three times/week. Exercise duration gradually increased during the following 4 weeks with the goal of reaching 45–60 min of exercise three days/week between weeks 4–6. Exercise intensity was measured continuously during the supervised exercise sessions via a Polar A10 Heart Rate Monitor chest strap and every 15 min via a rating of perceived exertion using the BORG scale (Borg, 1982). Participants were encouraged to maintain moderate intensity, equivalent to 60–75% of their age-predicted maximal heart rate. This duration, frequency, and intensity of aerobic exercise were maintained throughout the 6 months. Additionally, home-based exercise was reported by their participants in weekly diaries. Adherence to the exercise intervention, measured by the total time exercise (supervised and home-based sessions), adherence to the supervised exercise sessions, measured by the total time of supervised exercise, and average exercise intensity achieved during the supervised exercise sessions.

Research staff with background and ACSM certification in exercise training supervised the exercise sessions and recorded adherence, intensity, and safety information. Participants could choose their exercise mode (e.g., treadmills, elliptical machines, stationary bicycles, or concept two rower).

### 2.4 Usual care (control) group

Women randomized to the usual care condition were instructed to continue their usual activities for six months. Exercise was not withheld nor limited in this group.

### 2.5 Blinding

All investigators and research staff conducting assessments were blinded to random group assignment. The study’s principal investigators (PIs), statisticians, and interventionists were not blinded.

### 2.6 Study outcomes

#### 2.6.1 MRI data acquisition and pre-processing

The methods used for the MRI data acquisition and pre-processing for this sample have been previously described (Donofry et al., 2022). MRI sequences were collected using a Siemens Verio 3T scanner (Siemens Medical Solutions USA, Inc., Malvern, PA, United States) outfitted with a 32-channel head coil. High-resolution T1-weighted anatomical volumes were acquired in the sagittal plane using a magnetization-prepared rapid gradient-echo (MPRAGE) sequence for each participant (256 slices, voxel dimensions 1 × 0.976 × 0.976 mm). We used Freesurfer version 6.0 (Fischl, 2012) for volumetric segmentation, cortical surface reconstruction, and parcellation to quantify the brain volumes of interest. We obtained left and right volumes from the hippocampus, cortical GM, cerebral white matter (WM), subcortical GM, and cerebral GM, and an estimate of total intracranial volume (eTIV). Further, we obtained left and right cortical thickness for the precentral, superior frontal, superior parietal, superior temporal, frontal pole, and mean cortical thickness throughout the whole brain. Right and left values were averaged to obtain a bilateral value for each region of interest (ROI), as our hypotheses were not hemisphere-specific. The ROIs were selected based on their implications in memory and executive function processes, which are regulated by the hippocampus and subdivisions of the prefrontal cortex.

#### 2.6.2 Cardiovascular fitness

A submaximal graded exercise test was used to measure aerobic capacity using a modified Balke protocol (Fitchett, 1985). The protocol involved walking at a pace between 2.0 and 4.0 miles per hour (mph) with increasing grade increments of 1% every minute. The test speed was self-selected by the participant in consultation with the exercise physiologist at increments of 0.5 mph within the 2.0–4.0 mph range. Prior to the test, height and body mass were recorded, and the participant was fitted with a mouthpiece and nose clip to collect expired air. The test was terminated if any of the following criteria were met: (i) the participant reached 85% of her age-predicted maximal heart rate (220–age), (ii) a Borg rating (Borg, 1982) of perceived exertion of 15 or greater in participants taking a beta blocker, (iii) the participant reached volitional exhaustion, or (iv) safety concerns about continuation of the test by the monitoring medical team. Oxygen consumption was analyzed using a ParvoMedics metabolic cart (Parvo Medics TrueOne 2400), which uses an external mixing chamber with a pneumotach. Prior to each test, gas analyzers and flow meters were calibrated. VO_2submax_ was defined as the oxygen consumption relative to body weight in kilograms (ml/kg/min) achieved at 85% of the age-predicted maximal heart rate, and data were extrapolated to estimate maximal oxygen consumption (estimated VO_2peak_). VO_2peak_ was defined as the highest VO_2_ value recorded during the submaximal test using 15-second timed interval averaging.

### 2.7 Statistical methods

The mean and standard deviation (SD) were used to describe the baseline characteristics of the sample. Baseline demographics, VO_2_ measurements, and brain volumetric and cortical thickness values were compared between the exercise and control groups using the two-sample Wilcoxon test.

We assessed the effect of the 6-month exercise intervention on brain volumes and cortical thickness using linear mixed modeling (LMM). Models included the fixed effect for the main effect of treatment (6-month aerobic exercise intervention, control), time (baseline−T1, month 6−T2), and their interaction, as well as covariates, including age and eTIV at baseline. We adjusted for brain size using the covariate approach, as this has been shown to be more effective than the proportion method at removing the confounding effects of eTIV (Pintzka et al., 2015; Sanfilipo et al., 2004). We also included random effects (random intercept) for individual participants to account for the within-individual correlation among repeated measurements at baseline and month 6. LMM allowed us to model the changes in brain volumes and cortical thickness measures as a function of both time and group while also including potential confounding variables (e.g., age, eTIV) in the model. The LMM was implemented using the *lmer4* package in R.

Simple and multiple linear regression models were used to test the relationship between cardiorespiratory fitness changes and brain morphology changes (volumetric and cortical thickness values) from baseline to 6-month follow-up. We further explored the effect of the intervention in the aerobic exercise group by analyzing the association between average time of supervised exercise (min/week) and average exercise intensity achieved during the supervised sessions (%) with changes in brain morphology. Results were presented unadjusted (Model 1) and adjusted by age and eTIV (Model 2). For the purpose of this pilot study, we maintained a standard threshold of p < 0.05 for exploratory purposes and for estimating effect sizes.

We then conducted sensitivity analyses to assess the robustness of the results. Outliers with values more than 4 standard deviations above the mean of the dependent or independent variables were excluded from the analysis. Additionally, one participant who did not complete the supervised exercise sessions was excluded from the analysis. Standardized regression coefficients, SEs, *t*-values, *p*-values, and R-squared values were reported for all linear regression models (Cohen, 1962, 1988).

All analyses were performed using R software (version R 4.3.2).

## 3 Results

Baseline characteristics of the sample (*N* = 28) are summarized in Table 1. No significant differences were found between groups in baseline brain volumetric or cortical thickness values (all *p* > 0.05), except for baseline middle orbitofrontal cortical thickness where the aerobic exercise group had larger thickness values than the control group (*p* = 0.002).

**TABLE 1:** Baseline demographic characteristics and outcome values overall and by the study arms.

Variable	Aerobic exercise group	Control group	Total sample	*p*-value
**n**	**16**	**12**	**28**	
Age (years)	63.18 (5.89)	62.67 (4.91)	62.96 (5.4)	0.411
Education (years)	16.60 (3.20)	15.20 (2.52)	15.96 (2.96)	0.109
BMI	31.00 (7.80)	33.90 (5.01)	32.30 (6.79)	0.202
eTIV (cm^3^)	1450 (107.70)	1430 (178.10)	1438 (139.47)	0.471
Baseline VO_2peak_ (ml kg^–1^ min^–1^)	17.18 (3.28)	16.18 (2.35)	16.76 (2.92)	0.134
Baseline time to exhaustion (min)	7.86 (3.49)	8.19 (2.91)	8 (3.2)	0.608
**Brain volume**				
Hippocampus (mm^3^)	7824.67 (662.67)	7779.94 (1200.13)	7805.50 (911.74)	0.875
Cortical GM (mm^3^)	442137.60 (25690.58)	443503.90 (33771.45)	442723.2 (32877.5)	0.914
Cerebral WM (mm^3^)	413626.00 (39594.87)	431844.10 (54142.81)	421433.76 (46363.33)	0.194
Subcortical GM (mm^3^)	53491.94 (3019.35)	54098.17 (5034.78)	53751.75 (3935.15)	0.363
Cerebral GM (mm^3^)	595712.10 (32062.99)	602916.90 (55255.92)	598799.88 (42757.66)	0.546
**Cortical thickness**				
Precentral gyrus (mm)	2.62 (0.10)	2.59 (0.11)	2.61 (0.1)	0.747
Superior frontal gyrus (mm)	2.76 (0.10)	2.72 (0.08)	2.74 (0.09)	0.183
Superior parietal gyrus (mm)	2.32 (0.11)	2.32 (0.08)	2.32 (0.09)	0.934
Superior temporal gyrus (mm)	2.73 (0.12)	2.71 (0.13)	2.72 (0.12)	0.535
Frontal pole (mm)	2.83 (0.16)	2.79 (0.13)	2.82 (0.15)	0.271
Lateral orbitofrontal (mm)	2.62 (0.10)	2.66 (0.11)	2.64 (0.11)	0.197
Middle orbitofrontal (mm)	2.41 (0.10)	2.33 (0.11)	2.38 (0.11)	0.002*
Mean cortical thickness (mm)	2.52 (0.07)	2.50 (0.77)	2.51 (0.07)	0.199

Values are presented as mean (standard deviation). BMI, body mass index; eTIV, estimated total intracranial volume.

*p-value is from the two-sample Wilcoxon rank-sum test. Significant level at *p* < 0.05.

The study’s adherence rate for the intervention group (*n* = 16) was 112.5%, with an average of 169 minutes of exercise per week accounting for supervised in-person and home-based exercise sessions. The adherence rate to the supervised exercise sessions was 68%; when excluding the participant who did not perform in-person exercise sessions, the adherence rate was 72%. The average exercise intensity was 73% of the age-predicted maximal heart rate, with an average of 12 RPE during the supervised sessions.

### 3.1 Group differences in neuroimaging metrics of brain health

Table 2 shows the changes in brain volume and cortical thickness values between T1 and T2 for each group. The exercise group had smaller decreases over time in several brain volume areas, including the hippocampus, cortical gray matter (GM), cerebral white matter (WM), and cerebral GM, compared to the control group. However, no significant statistical time × group interactions were found in any volumetric measures (all *p* ≥ 0.05). Further, no statistically significant time × group interactions were found in the precentral, superior frontal, superior parietal, superior temporal, frontal pole, and mean cortical thickness (all *p* ≥ 0.05). The results remained the same after excluding the participant who did not complete supervised exercise sessions (see Supplementary Table 1).

**TABLE 2:** Changes in brain volume and cortical thickness from baseline to 6-month follow-up for both trial conditions.

	Change from baseline (Mean ± SD)		Statistical results (2-way interaction: group x time)						
**Cardiorespiratory fitness**	**Aerobic exercise** ** (*n* = 16)**	**Control (*n* = 12)**	**Between-group SMD** **(95%L, 95%U)**	**Estimate**	**SE**	**t**	**p**	**95% LCI**	**95% UCI**
VO_2peak_ (ml kg^–1^ min^–1^)	0.609 ± 1.860	0.492 ± 1.840	0.061 ( − 0.665, 0.786)	0.275	0.725	0.380	0.710	− 1.130	1.710
Time to exhaustion (min)	2.640 ± 3.280	1.040 ± 2.390	0.543 ( − 0.188, 1.260)	0.176	0.117	1.510	0.140	− 0.495	0.408
**Brain volume**									
Hippocampus (mm^3^)	−24.281 ± 109.386	−24.592 ± 77.845	0.003 (−0.706, 0.712)	0.310	37.156	0.008	0.993	−0.723	0.730
Cortex volume (mm^3^)	−818.182 ± 4294.181	−1557.422 ± 8250.905	0.107 (−0.638, 0.850)	739.240	2398.277	0.308	0.760	−3950.188	5428.668
Cerebral WM (mm^3^)	−664.616 ± 4164.975	−1994.049 ± 2215.293	0.385 (−0.322, 1.080)	1329.433	1327.506	1.001	0.326	−1266.282	3925.149
Subcortical GM (mm^3^)	−253.688 ± 468.704	−206.250 ± 389.675	−0.107 (−0.823, 0.612)	−47.437	166.888	−0.284	0.778	−373.760	278.885
Cerebral GM (mm^3^)	−1756.745 ± 5483.081	−1790.922 ± 9612.165	0.004 (−0.738, 0.746)	34.177	2868.800	0.012	0.991	−5575.279	5643.634
**Cortical thickness**									
Precentral gyrus (mm)	−0.005 ± 0.044	0.007 ± 0.055	−0.226 (−0.962, 0.514)	−0.012	0.019	−0.625	0.537	−0.048	0.025
Superior frontal gyrus (mm)	−0.011 ± 0.027	−0.004 ± 0.034	−0.199 (−0.934, 0.540)	−0.006	0.012	−0.549	0.588	−0.029	0.016
Superior parietal gyrus (mm)	0.001 ± 0.040	−0.015 ± 0.021	0.021 (−0.716, 0.756)	−0.001	0.018	−0.057	0.955	−0.025	0.037
Superior temporal gyrus (mm)	−0.010 ± 0.030	−0.004 ± 0.052	−0.138 (−0.879, 0.608)	−0.006	0.016	0.392	0.698	−0.025	0.037
Frontal pole (mm)	0.000 ± 0.096	−0.003 ± 0.080	0.030 (−0.687, 0.747)	0.003	0.034	0.081	0.936	−0.064	0.069
Lateral orbitofrontal (mm)	0.006 ± 0.040	−0.007 ± 0.044	0.294 (−0.444, 1.030)	0.013	0.016	0.803	0.429	−0.018	0.044
Middle orbitofrontal (mm)	−0.009 ± 0.042	−0.010 ± 0.066	0.025 (−0.716, 0.765)	0.001	0.020	0.380	0.710	−1.130	1.710
Mean thickness (mm)	−0.004 ± 0.021	−0.002 ± 0.036	−0.052 (−0.793, 0.691)	−0.002	0.011	1.510	0.140	−0.495	0.408

Sample included *n* = 28. GM, gray matter; WM, white matter; SMD, standardized mean difference; LCI, lower confidence interval;UCI, upper confidence interval; SE, standard error estimates. Significant level of 0.05.

### 3.2 Associations between changes in neuroimaging metrics and cardiovascular fitness changes

No significant associations were found between percentage change over the course of the intervention in submaximal VO_2peak_ and percent change in brain volume or cortical thickness (all *p* ≥ 0.05; see Supplementary Table 2). Additionally, no associations were found between percentage change in time to exhaustion and percentage change in brain volume or cortical thickness (all *p* ≥ 0.05; see Supplementary Table 2).

**FIGURE 1:**
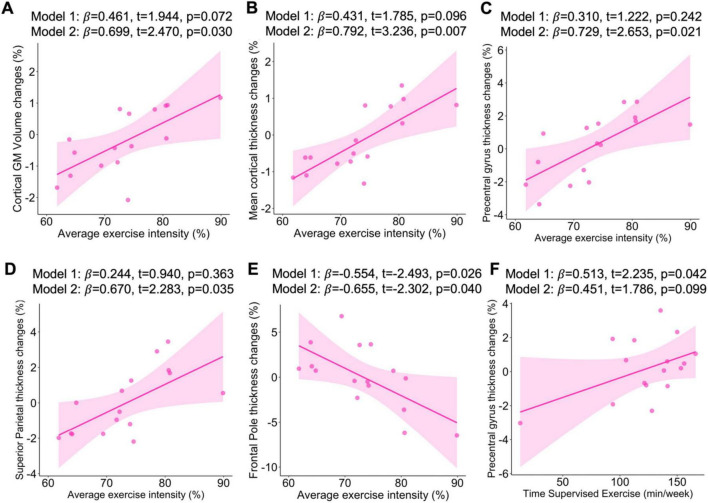
Associations of average exercise intensity with change in cortical GM volume **(A)**, mean thickness **(B)**, precentral gyrus thickness **(C)**, superior parietal thickness **(D)**, frontal pole thickness **(E)**. Associations of average time supervised exercise with change in precentral gyrus thickness **(F)**. Sample included *n* = 16. β, standardized regression coefficient; Significant level at *p* < 0.05. Model 1: Unadjusted analyses; Model 2: Analyses were adjusted for age and estimated total intracranial volume.

When examining the 16 participants assigned to the aerobic exercise group, we found that a greater average percent exercise intensity achieved over the 6-month period was associated with a greater percentage increase in cortical GM volume (*p* = 0.030, *R*^2^ = 0.413; see Figure 1A), as well as a greater increase in mean cortical thickness (*p* = 0.007, *R*^2^ = 0.561, see Figure 1B), precentral gyrus thickness (*p* = 0.021, *R*^2^ = 0.448; see Figure 1C), and superior parietal lobule thickness (*p* = 0.035, *R*^2^ = 0.421; see Figure 1D), after adjusting for age and eTIV. In contrast, and inconsistent with our hypotheses, higher percent exercise intensity was associated with greater decrease in frontal pole thickness even after adjusting by age and eTIV (*p* = 0.040, *R*^2^ = 0.407; see Figure 1E). Additionally, we found that greater average time of supervised exercise (min/week) was associated with a greater percentage increase in precentral gyrus thickness in our unadjusted model (*p* = 0.042, *R*^2^ = 0.210; see Figure 1F) and with a lower percentage change in superior temporal thickness in our unadjusted model when excluding the participants who did not complete the intervention (*p* = 0.008, *R*^2^ = 0.263; see Supplementary Figure 1), which persisted after adjusting by age and eTIV (*p* = 0.017, *R*^2^ = 0.437; see Supplementary Figure 1).

## 4 Discussion

We examined the effects of a 6-month moderate-intensity aerobic exercise intervention on several metrics of brain morphology in women treated for breast cancer. Contrary to our predictions, there was no evidence of an exercise intervention effect on hippocampal, cortical GM, cerebral WM, subcortical GM, or cerebral GM volumes, and no effect of the intervention on cortical thickness. Similarly, we found no associations between exercise-related increases in cardiorespiratory fitness and increases in brain morphology. However, importantly, a higher exercise intensity achieved during the supervised sessions was associated with greater increases in cortical GM, mean cortical thickness, precentral gyrus, and superior parietal thickness post-intervention. In further support of the idea that adherence to the intervention matters, we found that total supervised exercise time was associated with greater thickness of the precentral gyrus after the intervention.

The lack of significant intervention effects might not be surprising given the small sample size and pilot nature of this study. In fact, most effect sizes of morphological brain changes resulting from exercise interventions that have been generated from meta-analyses suggest modest effects that likely require > 100 participants to reliably detect an effect. Additionally, the subsample included in these analyses showed small changes in cardiovascular fitness after the intervention suggesting that if changes in cardiovascular fitness mediates the effect of exercise on brain morphology, then we would expect small-to-negligible effects on brain morpohology. However, whether improvements in cardiorespiratory fitness drives an effect on the brain remains a matter of continued debate in the field. Yet, when examining the mean differences of changes over time between both groups, we observed smaller decreases in brain volumes (e.g., hippocampus, cortical GM, cerebral WM, subcortical GM, and cerebral GM) in the aerobic exercise group compared to the control group. Although the trends did not reach statistical significance, they suggest that a well-powered study might detect differences between groups over the course of the intervention. It is also possible that the average age of our sample contributed to reduced effect sizes. A recent meta-analysis reported a positive impact of exercise on hippocampal volume in participants aged 65 or older but not in participants younger than 65 (Wilckens et al., 2021). Our sample was, on average, 62.96 years of age. Although it is possible that the benefits of exercise might be greater in populations with either cognitive impairment or accelerated aging (e.g., breast cancer patients), these results suggest that the exercise intervention might show a different magnitude of response to brain morphology as a function of age. However, the moderating effect of age on the association between physical exercise and brain morphology is unclear due to the heterogeneity among intervention designs (i.e., duration, intensity, or type of exercise). Future research should examine this further.

Cardiorespiratory fitness has been consistently linked to better neurocognitive performance in numerous populations, including women treated for breast cancer (Campbell et al., 2020; Chaddock-Heyman et al., 2015; Koevoets et al., 2023; Koevoets et al., 2022; Ye Xin-Xin et al., 2022). We previously reported in this sample at baseline that higher cardiorespiratory fitness was associated with elevated resting state functional connectivity (rsFC) of the hippocampus with prefrontal brain regions (Lesnovskaya et al., 2023). Therefore, although fitness levels have been extensively linked to neurocognitive improvements, there is a lack of understanding of this relationship in vulnerable populations such as women with breast cancer.

Finally, despite the lack of a statistically significant effect of the exercise intervention on brain volumes and cortical thickness, our results support the hypothesis that exercise intensity could be driving exercise effects on brain morphology. Specifically, higher exercise intensity was associated with greater increases in cortical GM and cortical thickness (e.g., precentral gyrus, superior parietal, and mean cortical thickness), reflecting moderate effect sizes (adjusted *R*^2^ from 0.26 to 0.46). Finding these effect sizes in this small pilot sample suggests that exercise intensity could play a key role, as others have previously indicated (Koevoets et al., 2023; Wilckens et al., 2021). However, the current findings must be interpreted with caution due to the small sample size and the pilot nature of this nested sub-study.

Strengths of the current pilot study include the randomized and rigorous nature of the exercise intervention, the length of the intervention (6 months), the achieved intensity of the intervention (60–75% maximal heart rate; 150 min/week), and type of intervention (moderate-intensity aerobic exercise) in a vulnerable sample such as women with breast cancer. Additionally, the intervention was supervised, and patients showed good average adherence to the program (72%). However, this nested sub-study presented several limitations. There were several challenges in recruiting this patient population for MRI pre-therapy, such as time constraints or incompatible seeds/implants related to breast biopsy procedures. However, although our sub-study sample size was small, the significant associations found between exercise intensity and percentage changes in brain morphology had moderate effect sizes. In conclusion, the current preliminary results indicate that the aerobic exercise intervention was not effective at increasing brain volumes and cortical thickness in women with breast cancer. However, the positive associations between average exercise intensity during the intervention and percentage change in brain morphology suggest that exercise may benefit this population by potentially reducing the vulnerability of the brain to the deleterious effects of breast cancer and its treatment. Future studies with more well-powered samples are needed to test these effects.

## Data Availability

The raw data supporting the conclusions of this article will be made available by the authors, without undue reservation.
