# Supplementary material for: Effects of a 6-month aerobic exercise intervention on brain morphology in women with breast cancer receiving aromatase inhibitor therapy: a sub-study of the EPICC trial
Source: Front Hum Neurosci. 2024 Oct 23;18:1443916. doi: 10.3389/fnhum.2024.1443916 (PMC11538074; doi:10.3389/fnhum.2024.1443916)
Supplement: Supplementary file 1 [file Data_Sheet_1.pdf]

1. Supplementary Material

Supplementary Table 1. Changes in brain volume and cortical thickness from baseline to 6-month follow-ups for both trial conditions

	Change from baseline (Mean ± SD)			Statistical results (2-way interaction: group x time)					
Cardiorespiratory fitness	**Aerobic exercise (n=15)**	**Control (n=12)**	**Between-group SMD (95%L, 95%U)**	**Estimate**	**SE**	**t**	**p**	**95% LCI**	**95% UCI**
VO_{2peak} (ml kg^{-1} min^{-1})	0.704±1.890	0.492±1.840	0.110 (–0.627, 0.844)	0.275	0.073	0.380	0.707	–1.130	1.710
Time to exhaustion (min)	2.650±3.390	1.040±2.390	0.533 (–0.209, 1.260)	1.760	1.170	1.510	0.143	–0.495	4.080
Brain volume									
Hippocampus (mm^3)	–24.100±113.00	–24.592±77.845	0.005 (–0.715, 0.725)	–0.478	348.430	–0.012	0.990	–75.610	74.538
Cortex volume (mm^3)	–941.000±4415.700	–1557.422±8250.905	0.089 (–0.660, 0.836)	–616.500	2476.000	–0.249	0.805	–5457.408	4224.504
Cerebral WM (mm^3)	–1171.400±3766.100	–1994.049±2215.293	0.257 (–0.463, 0.971)	–822.700	1231.000	–0.668	0.510	–43229.364	1584.010
Subcortical GM (mm^3)	–267.500±481.800	–206.250±389.675	–0.136 (–0.862, 0.594)	61.280	171.800	0.357	0.724	–2746.121	397.179
Cerebral GM (mm^3)	–1894.400±5646.800	–1790.922±9612.165	–0.013 (–0.759, 0.734)	103.400	2963.000	0.035	0.972	–5688.516	5895.412
Cortical thickness									
Precentral gyrus (mm)	–0.001±0.042	0.007±0.055	–0.148 (-0.891, 0.599)	0.075	0.0187	0.403	0.690	–0.029	0.044
Superior frontal gyrus (mm)	–0.008±0.027	–0.004±0.034	–0.118 (-0.861, 0.627)	0.004	0.012	0.321	0.751	–0.019	0.027
Superior parietal gyrus (mm)	0.001±0.041	–0.015±0.021	–0.0.45 (-0.698, 0.787)	–0.002	0.002	-0.123	0.903	–0.026	0.037
Superior temporal gyrus (mm)	–0.010±0.031	–0.004±0.052	–0.133 (-0.879, 0.616)	0.006	0.016	0.370	0.714	–0.026	0.038
Frontal pole (mm)	–0.006±0.096	–0.003±0.080	–0.038 (-0.766, 0.691)	0.003	0.035	0.099	0.922	–0.064	0.071
Lateral orbitofrontal (mm)	0.004±0.040	–0.007±0.044	0.254 (-0.491, 0.994)	–0.011	0.016	-0.682	0.502	–0.043	0.021
Middle orbitofrontal (mm)	–0.009±0.042	–0.010±0.066	0.028 (-0.718, 0.773)	–0.016	0.021	-0.076	0.934	–0.043	0.040
Mean thickness (mm)	–0.003±0.021	–0.002±0.036	–0.014 (-0.761, 0.733)	0.024	0.025	0.979	0.337	–0.024	0.072

Sample included n = 27, excluding the participant with low adherence in the aerobic exercise group. **Abbreviations:** GM, grey matter; WM, white matter; SDM, standardized mean difference. Significant level at $p < 0.05$.

Supplementary Table 2. Association of percentage change in VO_{2peak} with the percentage change in brain volume and cortical thickness; and association of percentage change in time to exhaustion with the percentage change in brain volume and cortical thickness.

	**Change VO_{2peak} (%) N=28**			**Change time to exhaustion (%) n=27**		
Variable	β	P value	R^2	β	P value	R^2
Hippocampus (%)						
Model 1	–0.116	0.558	–0.025	–0.003	0.638	–0.031
Model 2	–0.160	0.414	0.068	0.001	0.750	0.038
Cortical GM (%)						
Model 1	-0.205	0.295	0.005	0.001	0.942	–0.040
Model 2	-0.211	0.315	–0.076	–0.001	0.999	–0.128
Cerebral WM (%)						
Model 1	0.134	0.496	–0.020	0.005	0.155	0.042
Model 2	0.153	0.466	–0.020	0.005	0.181	–0.022
Subcortical GM (%)						
Model 1	–0.176	0.372	–0.006	–0.002	0.615	–0.029
Model 2	–0.219	0.284	–0.017	–0.000	0.951	–0.073
Cerebral GM (%)						
Model 1	–0.322	0.095	0.069	0.000	0.872	–0.039
Model 2	–0.319	0.122	–0.008	0.000	0.919	–0.117
Cortical thickness						
Precentral gyrus (%)						
Model 1	–0.158	0.422	–0.013	–0.005	0.543	–0.024
Model 2	–0.134	0.524	–0.086	–0.007	0.432	–0.079
Superior frontal gyrus (%)						
Model 1	–0.047	0.811	–0.036	–0.002	0.580	–0.027
Model 2	–0.054	0.797	–0.122	–0.003	0.583	–0.115
Superior parietal gyrus (%)						
Model 1	–0.283	0.144	0.045	–0.003	0.714	–0.034
Model 2	–0.293	0.158	–0.020	–0.000	0.839	–0.098
Superior temporal (%)						
Model 1	**–0.379**	**0.046**	**0.111**	0.002	0.817	–0.038
Model 2	–0.346	0.083	0.071	–0.001	0.867	–0.042
Frontal pole (%)						
Model 1	0.089	0.482	–0.030	0.010	0.480	–0.019
Model 2	0.131	0.518	–0.008	0.002	0.234	0.022
Lateral orbitofrontal (%)						
Model 1	0.000	0.999	–0.038	0.004	0.589	–0.028
Model 2	0.019	0.926	–0.082	0.001	0.873	–0.083
Medial orbitofrontal (%)						
Model 1	0.198	0.312	0.002	0.008	0.450	–0.016
Model 2	0.189	0.367	–0.067	0.001	0.279	–0.054
Mean cortical thickness (%)						
Model 1	–0.267	0.169	0.036	–0.001	0.908	–0.040
Model 2	–0.272	0.193	–0.043	–0.000	0.925	–0.120

Statically significant values are shown in bold. **Abbreviations:** GM, grey matter; WM, white matter; β, standardized regression coefficient; R^2, Adjusted R squared; Significance level at $p \leq 0.05$. Model 1: Unadjusted analyses; Model 2: Analyses were adjusted for age and estimated total intracranial volume.

Supplementary Table 3. Association of average exercise intensity with percentage change in brain volume and cortical thickness in the aerobic exercise group.

	**Exercise intensity (%) n=16**		
Variable	β	P value	R^2
Hippocampus (%)			
Model 1	0.010	0.970	–0.071
Model 2	0.192	0.600	–0.170
Cortical GM (%)			
Model 1	0.461	0.072	0.156
Model 2	**0.699**	**0.030**	**0.267**
Cerebral WM (%)			
Model 1	–0.062	0.818	–0.067
Model 2	–0.165	0.657	–0.208
Subcortical GM (%)			
Model 1	0.215	0.423	–0.022
Model 2	0.383	0.293	–0.113
Cerebral GM (%)			
Model 1	0.368	0.161	0.073
Model 2	0.596	0.090	0.044
Cortical Thickness			
Precentral gyrus (%)			
Model 1	0.310	0.242	0.032
Model 2	**0.728**	**0.021**	**0.309**
Superior frontal (%)			
Model 1	0.332	0.209	0.047
Model 2	0.497	0.090	0.331
Superior parietal gyrus (%)			
Model 1	0.244	0.363	–0.008
Model 2	**0.670**	**0.035**	**0.276**
Superior temporal (%)			
Model 1	0.294	0.268	0.021
Model 2	0.303	0.407	–0.141
Frontal pole (%)			
Model 1	**–0.554**	**0.026**	**0.258**
Model 2	**–0.655**	**0.040**	**0.259**
Lateral orbitofrontal (%)			
Model 1	0.470	0.066	0.166
Model 2	0.355	0.260	0.174
Medial orbitofrontal (%)			
Model 1	–0.055	0.839	–0.068
Model 2	–0.015	0.961	0.197
Mean cortical thickness (%)			
Model 1	0.431	0.096	0.127
Model 2	**0.792**	**0.007**	**0.450**

Statistically significant values are shown in bold. Sample included n = 16. **Abbreviations:** GM, gray matter; WM, white matter; β, standardized regression coefficient; R^2, Adjusted R squared; Significance level at $p < 0.05$. Model 1: Unadjusted analyses; Model 2: Analyses were adjusted for age and estimated total intracranial volume.

Supplementary Table 4. Average supervised exercise time is associated with changes in brain volume and cortical thickness in the aerobic exercise group.

	**Time supervised exercise (min/week) n=16**			**Time supervised exercise (min/week) n=15**		
DVariable	β	P value	R^2	β	P value	R^2
Hippocampus (%)						
Model 1	−0.176	0.515	−0.038	−0.316	0.251	0.031
Model 2	−0.218	0.467	−0.144	−0.338	0.252	−0.077
Cortex volume (%)						
Model 1	−0.252	0.346	−0.003	−0.259	0.351	−0.004
Model 2	−0.333	0.239	0.019	−0.275	0.329	−0.002
Cerebral WM (%)						
Model 1	−0.285	0.285	0.016	0.278	0.316	0.006
Model 2	−0.274	0.363	−0.143	0.274	0.367	−0.173
Subcortical GM (%)						
Model 1	−0.352	0.182	0.061	−0.444	0.098	0.135
Model 2	−0.425	0.147	−0.021	−0.464	0.107	0.038
Cerebral GM (%)						
Model 1	−0.369	0.159	0.075	−0.486	0.066	0.177
Model 2	−0.434	0.138	−0.014	−0.502	0.078	0.077
Cortical thickness						
Precentral gyrus (%)						
Model 1	**0.513**	**0.042**	**0.210**	0.180	0.520	−0.042
Model 2	0.451	0.099	0.134	0.160	0.593	−0.166
Superior frontal (%)						
Model 1	0.456	0.076	0.152	0.262	0.346	−0.003
Model 2	0.401	0.097	0.324	0.262	0.303	0.190
Superior parietal gyrus (%)						
Model 1	−0.028	0.916	−0.071	−0.197	0.481	−0.035
Model 2	−0.262	0.572	−0.037	−0.241	0.395	−0.026
Superior temporal (%)						
Model 1	−0.375	0.153	0.079	**−0.652**	**0.008**	**0.381**
Model 2	−0.360	0.222	−0.063	**−0.639**	**0.017**	**0.283**
Frontal pole (%)						
Model 1	−0.037	0.891	−0.070	0.275	0.321	0.005
Model 2	−0.168	0.555	−0.037	0.226	0.396	0.096
Lateral orbitofrontal (%)						
Model 1	−0.191	0.480	−0.033	−0.114	0.685	−0.063
Model 2	−0.036	0.892	0.079	−0.057	0.832	0.042
Medial orbitofrontal (%)						
Model 1	−0.166	0.538	−0.042	−0.305	0.268	0.024
Model 2	−0.326	0.171	0.317	−0.344	0.142	0.350
Mean cortical thickness (%)						
Model 1	0.016	0.953	−0.071	−0.266	0.339	−0.001
Model 2	−0.091	0.747	−0.019	−0.291	0.297	0.027

Statistically significant values are shown in bold. Sample included n = 16. Sensitivity analysis, excluding the participant with low adherence, included sample n = 15. **Abbreviations:** GM, grey matter; WM, white matter; β, standardized regression coefficiet; R^2, Adjusted R squared; Significant level at $p < 0.05$. Model 1: Unadjusted analyses; Model 2: Analyses were adjusted for age and estimated total intracranial volume.

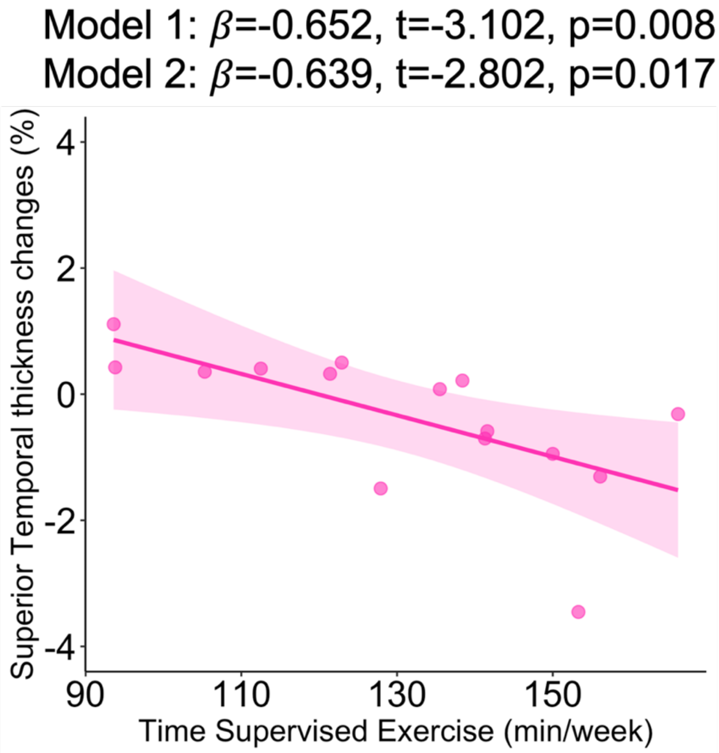

Supplementary Figure 1. Associations of average time supervised exercise (min/week) and percentage change in superior temporal gyrus after excluding the participants with low adherence. Sample included n = 15. β, standardized regression coefficient; Significance level at $p < 0.05$. Model 1: Unadjusted analyses; Model 2: Analyses were adjusted for age and estimated total intracranial volume.
